# A novel asynchronous access method with binary interfaces

**DOI:** 10.1186/1743-0003-5-24

**Published:** 2008-10-29

**Authors:** Jorge Silva, Jorge Torres-Solis, Tom Chau, Alex Mihailidis

**Affiliations:** 1 Komodo OpenLab, Toronto, Canada; 2 Bloorview Research Institute, Bloorview Kids Rehab, University of Toronto, Canada; 3 Institute of Biomaterials and Biomedical Engineering, University of Toronto, Canada; 4 Intelligent Assistive Technologies and Systems Lab, University of Toronto, Canada

## Abstract

**Background:**

Traditionally synchronous access strategies require users to comply with one or more time constraints in order to communicate intent with a binary human-machine interface (e.g., mechanical, gestural or neural switches). Asynchronous access methods are preferable, but have not been used with binary interfaces in the control of devices that require more than two commands to be successfully operated.

**Methods:**

We present the mathematical development and evaluation of a novel asynchronous access method that may be used to translate sporadic activations of binary interfaces into distinct outcomes for the control of devices requiring an arbitrary number of commands to be controlled. With this method, users are required to activate their interfaces only when the device under control behaves erroneously. Then, a recursive algorithm, incorporating contextual assumptions relevant to all possible outcomes, is used to obtain an informed estimate of user intention. We evaluate this method by simulating a control task requiring a series of target commands to be tracked by a model user.

**Results:**

When compared to a random selection, the proposed asynchronous access method offers a significant reduction in the number of interface activations required from the user.

**Conclusion:**

This novel access method offers a variety of advantages over traditionally synchronous access strategies and may be adapted to a wide variety of contexts, with primary relevance to applications involving direct object manipulation.

## Background

Many Disabled individuals require custom interfaces that enable them to access the devices they may wish to control. When appropriately designed, such interfaces take advantage of the user's known abilities, while eliminating reliance on onerous operational requirements. Thus, the design of appropriate user interfaces for Disabled individuals involves a process of understanding the needs, challenges and abilities of each user. In order to facilitate this process, it is necessary to count on widely available and highly adaptable tools that may be customized and combined in order to obtain the most appropriate solutions in each case. One such tool is the binary interface (commonly represented as a button or a switch), which, due to its simplicity and adaptability, has become a ubiquitous resource to overcome barriers to access for Disabled people.

A binary interface is formally defined as a device that may present only one of two distinct and stable states at any given time (e.g., on/off), which may be used to convey information between two entities [1]. Moreover, according to basic principles of information theory, binary interfaces are in fact the simplest possible means through which a user may communicate intent, since they represent the basic unit of information, namely, the binary digit or bit [2]. Therefore, binary interfaces may also be termed minimal interfaces. Minimal interfaces for Disabled users include other means of communication characterized by a low information storage (i.e., memory) capacity, this is the case, for example, with most brain-computer interfaces (BCI) currently available [3,4].

### The problem of binary access

In order to communicate intent through a binary interface, a user must be able to intentionally determine, whenever necessary, which of the two possible states the interface should present. Thus, for example, in the case of a button, the user must be able to intentionally perform the mechanical actions required to press and release the button. Other binary interfaces may, for example, exploit the user's ability to produce a gesture [5] or blink [6] at will.

More recently, researchers have explored the detection of voluntary changes in physiological activity, such as brain [7] or electrodermal activity [8], in order to obtain a few distinct and repeatable patterns that, similarly to binary interfaces, may be used to communicate intent. These novel approaches may provide a means of access for those users whose intent may not be understood otherwise. Some of these physiological interfaces, although still minimal, are capable of respresenting more than 1 bit of information at once, however, due to a variety of design, measurement and contextual challenges, their implementation is generally simpler and more effective when only a binary mode of use is required.

**Figure 1:**
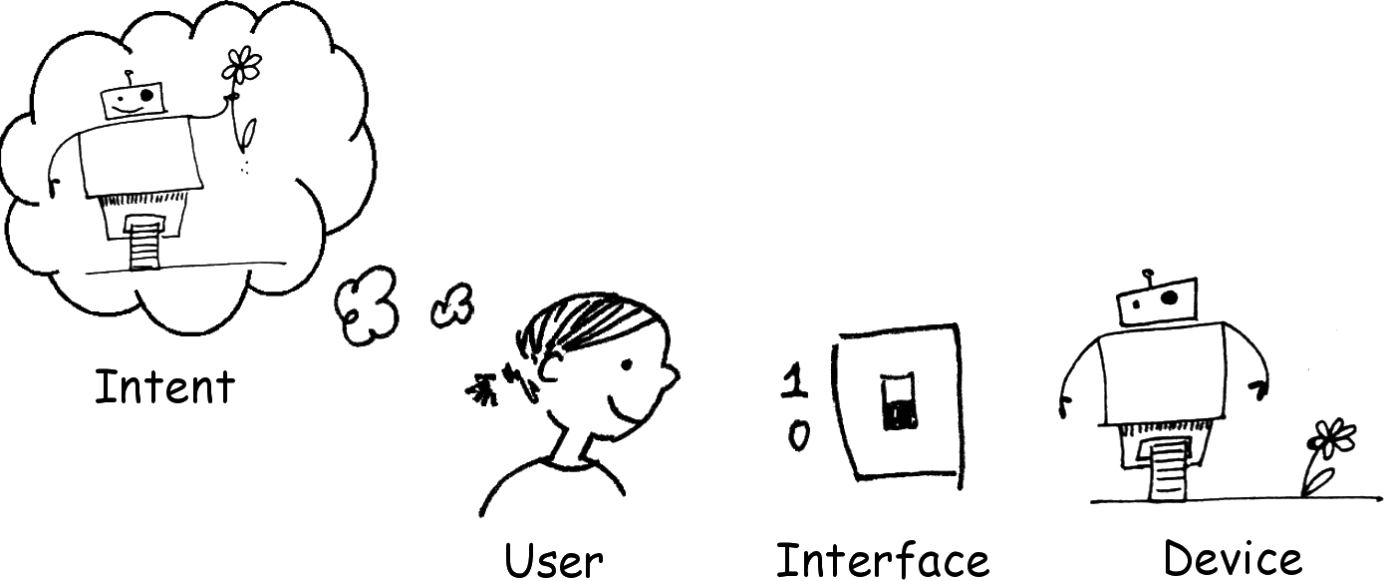
**The problem of access with binary interfaces**. A user is required to communicate intent, by means of a binary interface, to a device capable of more than two outcomes.

In spite of all these advantages, binary interfaces also present significant limitations that preclude their use in a wide variety of access and control applications. Evidently, the binary nature of these interfaces makes them an ideal solution for the control of devices with intentional spaces that present only a dyad of possibilities (e.g., close-open, up-down, etc.) However, when access to more than two distinct outcomes is required for the successful control of a device, the limitations of the binary interface become immediately apparent. Figure 1 depicts this dilemma where a user is required to control a complex device by means of a binary interface.

#### Protocol-based binary access

**Figure 2:**
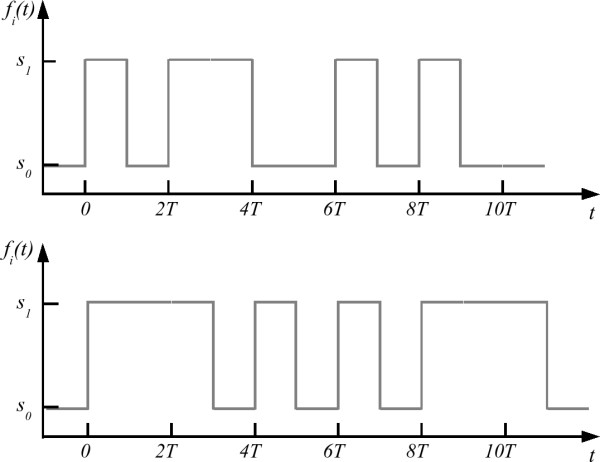
**Sample state sequences *f*_*i*_(*t*) used to communicate a particular message through a binary channel**. The top trace represents the hexadecimal number 9A_16 _in the RS232 serial communication protocol. The bottom trace represents the letter '*X*' in Morse code.

Consider the set *S *= {*s*_0_, *s*_1_, *s*_2_,...,*s*_*ς*-1_} containing all *ς *states available in a typical interface. Note that, with a binary interface, *ς *= 2. This interface, is used to access the set *C *= {*c*_0_, *c*_1_, *c*_2_,..., *c*_*κ*-1_} of size *κ*, containing all possible outcomes available for selection. The initial limitation of the case *κ *> *ς *is typically overcome by the implementation of a time-bound protocol that enables the generation of a new set *S*_*T *_= {*f*_0_(*t*), *f*_1_(*t*), *f*_2_(*t*),...,*f*_*κ*-1_(*t*)} where each element *f*_*i*_(*t*) ∈ *S*_*T *_is a time-dependent function composed by a unique sequence of channel states *s*_*i *_∈ *S *with duration *T *. This time-based coding enables the direct mapping of each member *f*_*i*_(*t*), of the newly created set of functions *S*_*T*_, to a unique message *c*_*i *_∈ *C*. Figure 2 shows two sample periodic state sequences *f*_*i*_(*t*) used to communicate messages through a binary interface (i.e., *ς *= 2). The top sequence represents the hexadecimal number 9A_*HEX *_as defined by the RS232 serial communication protocol. The bottom sequence represents the letter '*X*' as defined by the Morse code. Evidently, there are significant similarities between early electronic communication challenges and the use of binary interfaces by Disabled users. These similarities were quickly identified by interface designers who transferred the application of time-bound communication protocols to the implementation of access solutions for the Disabled. In fact, Morse-based communication and computer access methods are still being actively researched [9,10].

There are, however, some significant disadvantages with the use of time-bound protocols in the control of a device by a human operator. These stem mainly from the fact that both the transmitting and the receiving end must comply with the protocol used in the communication process. This requires users to either memorize all pairs {*f*_*i*_(*t*), *c*_*i*_} mapping every device outcome *c*_*i *_∈ *C *to its corresponding sequence *f*_*i*_(*t*) ∈ *S*_*T*_, or learn the time-coding rule *g*(*t*) : *f*_*i*_(*t*) → *c*_*i *_that may be used to generate the *i*-th sequence *f*_*i*_(*t*) ∈ *S*_*T *_corresponding to the desired outcome *c*_*i *_∈ *C*. Evidently, depending on individual abilities, this requirement will affect different users to varying degrees. However, the number *κ *of device outcomes that can be made available to the user will be largely limited by the user's memory capacity as well as the complexity of the protocol. Therefore, this requirement will impose, in all cases, an upper boundary *κ*_*max *_on *κ *(i.e., *κ *≤ *κ*_*max*_).

#### Scanning-based binary access

In order to maximize the value of *κ*_*max*_, feedback systems with varying degrees of complexity have also been developed. Some of these are designed to remind the user of the protocol's guidelines [11], while others, relying on periodic sensory cues, may completely eliminate the need for memorization [12]. This latter category includes all scanning access methods, commonly used by Disabled people nowadays. With scanning methods, all possible outcomes are presented to the user, at once, by means of a sensory pathway (usually visual and/or auditory). During operation, the outcomes are automatically highlighted, one by one, at a given rate according to the user's abilities. In order to indicate intent, users are required to activate the binary interface whenever their desired outcome is highlighted. This process results in the generation of time-dependent sequences *f*_*i*_(*t*) similar to the ones depicted in Figure 2. However, in contrast to the protocols formerly described, there is far more tolerance for variance in the period *T *during which the state of the interface must be maintained. Furthermore, because scanning methods rely mostly on the feedback information about the state of the scanning process presented to the user, there are usually sequences *f*_*i*_(*t*) ∈ *S*_*T *_that correspond to more than one outcome *c*_*i *_∈ *C*. These characteristics make scanning methods accessible to a wider variety of users and extend the range of potential applications beyond those available with the more formal protocols described above. However, scanning access methods still present a significant drawback: the timing of the interaction is controlled by an automatic agent, not by the user. Thus, even after the user has already decided on the intended outcome, (s)he must still wait until this outcome is highlighted by the automated scanning process in order to communicate the intention. A variety of strategies have been proposed to optimize this process and therefore reduce the time required for the intended outcome to be selected [12,13], however, the basic principle remains the same. As a result, with scanning access methods, it is time, rather than memory capacity or protocol complexity, that limits the maximum number, *κ*_*max*_, of device outcomes that can be made accessible to the user.

#### Synchronous vs. asynchronous binary access

Because of the external time constraints imposed on the user, both protocol-based and scanning-based access methods are more generally defined as *synchronous *in the study of human machine interfaces (HMI). Within this field, a synchronous access strategy may be defined as a method that requires users to comply with one or more time constraints in order to communicate intent with a minimal interface. This implies that, with synchronous access strategies, there will always be an additional delay in the process of selection of the intended outcome.

Conversely, *asynchronous *access methods do not place any time constraints on the users. Thus, users may initiate control of the device at any time without having to wait for external cues. Furthermore, no protocols are necessary because a single interface activation is sufficient to transmit a full unambiguous message to the device under control. Therefore, there is no additional delay in the selection of the intended outcome. When using binary interfaces, this is easily achievable when the intention space only presents two possibilities. That is, when the number of possible device outcomes is *κ *= 2.

Consider, for example, a wall switch with states *S *= {*s*_0 _: UP, *s*_1 _: DOWN} used to select one of the two possible outcomes *C *= {*c*_0 _: ON, *c*_1 _: OFF} of a light bulb. In this case, it is possible to map directly each outcome *c*_*i *_∈ *C *with a particular interface state *s*_*i *_∈ *S *in order to establish a suitable control strategy:

(1) $$c_{i}=\{\begin{array}{ll} c_{0}:\text{ON} & \text{if }s_{0}:\text{UP}\\ c_{1}:\text{OFF} & \text{if }s_{1}:\text{DOWN} \end{array}$$

According to Equation (1), every time the position of the wall switch changes, the behavior of the light bulb will change accordingly. Thus, a single change in the wall switch represents a full, unambiguous command sent to the light bulb, allowing the latter to respond immediately.

It has always been assumed that this kind of asynchronous access is impossible in cases where the number *κ *of outcomes *C *required to control a device is greater than the number *ς *of states *S *available in the interface. However, the method presented in this paper may be used with minimal interfaces presenting as few as *ς *= 2 stable states, in order to access, asynchronously, sets of device outcomes of any size *κ *∈ {2, 3, 4,...}. This includes those belonging to analog, as well as multidimensional domains, such as the movement parameters of an object in a 3-dimensional space. As a result, a variety of activities not typically available to Disabled users, may now be made accessible to them.

In the following sections, we provide details on the mathematical development of the proposed method for asynchronous access, the necessary guidelines for its implementation, and an initial evaluation based on a simulated control task. Our concluding remarks and suggestions for future work, are summarized in latter sections.

## A new method for asynchronous binary access

To present the proposed method for asynchronous access, we will initially focus on the case where a binary interface must be used to access a set of outcomes of arbitrary size, in order to control a particular device or perform a specific task. It is important to note that this analysis was originally prompted by the solution of a specific access challenge, namely, the development of an appropriate strategy to facilitate *binary navigation control*. In the context of disability engineering, binary navigation control consists of enabling users to voluntarily define and/or modify the motion parameters of an object in space, at any time, by means of a binary interface. Binary navigation control is thus required to enable most activities involving object manipulation with binary interfaces (e.g., single-switch drawing). Many such activities are currently inaccessible to binary and other minimal interface users. For example, when defining suitable alternatives for computer access, Shein (1997) described single-switch, computer-aided drawing as an exceptionally challenging activity that, unlike many other computer-related tasks, may not be broken into predictable sequences accessible through standard synchronous methods [14].

Consider a user who attempts to employ a single button (single-switch) to access a device requiring a set *C *of *κ *> 2 outcomes. The button, in turn, presents only *ς *= 2 possible states *S *= {*s*_0 _: released, *s*_1 _: pressed}. Thus, a simple mapping strategy such as the one shown in Equation (1) may not be used.

Initially, we may define the transition from state *s*_0 _to state *s*_1 _(i.e., a button press) as an intentional, user-prompted change in the interface. We will call this event ϵ. For the sake of simplicity, we will assume that the opposite transition (i.e., a button release) is not an intentional event and thus, will not represent a change in the interface.

According to the principle of asynchronous access described above, every time ϵ occurs, the behavior of the device must be changed. In other words, a new device outcome *c *∈ *C *must be selected. Note that this principle suggests that the event ϵ is only necessary when the behavior of the device is unacceptable to the user since this would be the only instance where a change in the behavior of the device would be welcome. Conversely, if the behavior of the device is already consistent with the user's intention, the event ϵ is not required. In other words, in our example, the button should be used to indicate the presence of unacceptable behaviors (i.e., errors) in the device through the intentional generation of events ϵ.

Let *n *be the count of consecutive events ϵ, and *c*_[*n*] _∈ *C *the device outcome chosen in response to the *n*-th occurrence of ϵ. The fundamental principle of asynchronous access may then be simply defined as:

(2) *c*_[*n*] _≠ *c*_[*n*-1]_

This principle states that when the *n*-th event ϵ occurs, the resulting device outcome *c*_[*n*] _must be different from the outcome *c*_[*n*-1] _preceding it. We call this principle a negative acknowledgement (NAK) signaling process because the user is required to activate the interface only when the device behaves erroneously. This term has been borrowed from the analogous error detection, out-of-band, signaling system for error control, often used in telecommunications [15], which, because of its simplicity, has been shown to reduce the communication costs (in terms of time and bandwidth) in environments with significant processing constraints [16].

### The exclusion mask

**Figure 3:**
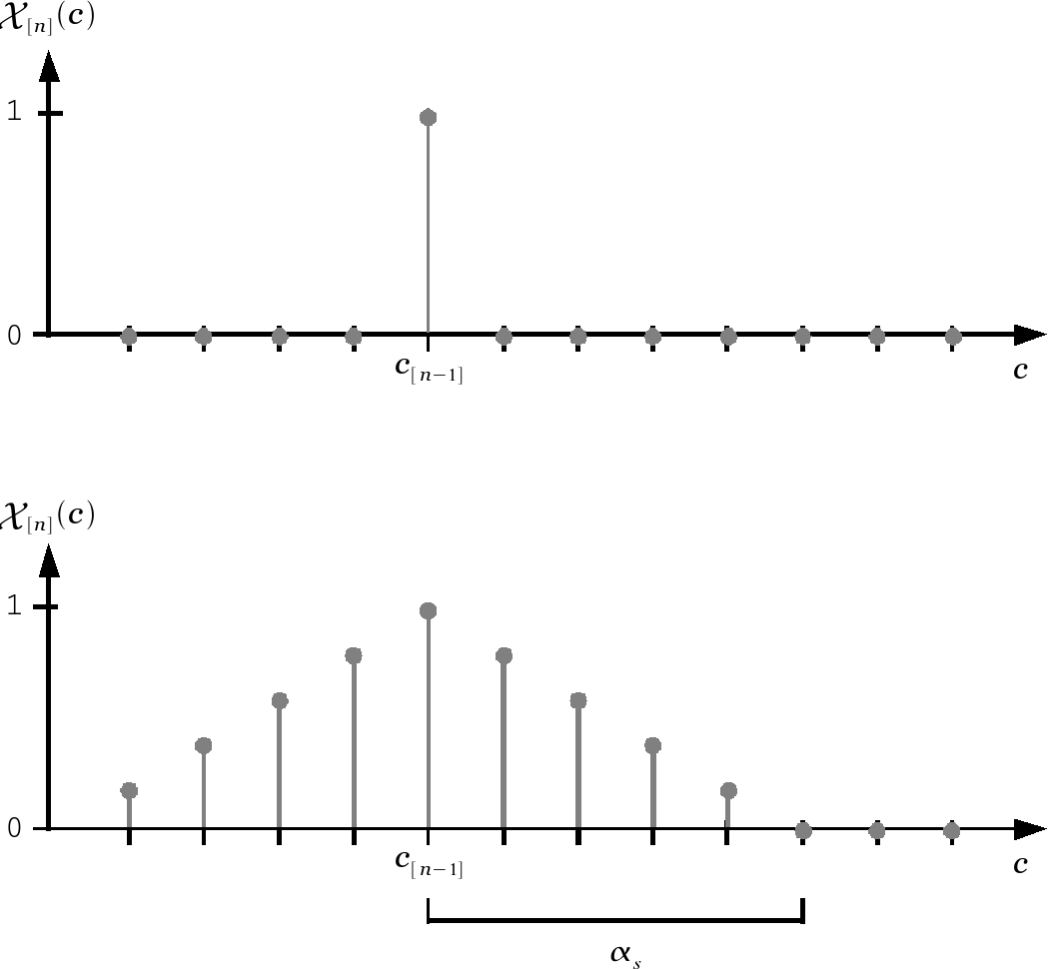
**Sample spatial exclusion masks $X_{\left[n\right]}$(*c*)**. The top mask represents the fundamental knowledge implied by the principle of asynchronous access. Because of its maximum value $X_{\left[n\right]}$(*c *= *c*_[*n*-1]_) = 1, the element *c*_[*n*-1] _cannot be chosen when selecting a new device outcome *c*_[*n*]_. The bottom mask represents the assumption that outcomes similar to *c*_[*n*-1] _should also be excluded from the selection of *c*_[*n*]_.

With the exception of Equation (2), there is no additional information that could help us determine, precisely, which of the remaining elements *c *≠ *c*_[*n*-1] _of *C *should be selected as the outcome *c*_[*n*]_. The top trace in Figure 3 shows an alternative graphical representation of this knowledge, which may be formally defined as

(3) $$X_{\left[n\right]}(c)=\{\begin{array}{cc} 1 & \text{if }c=c_{\left[n-1\right]}\\ 0 & \text{if }c\neq c_{\left[n-1\right]} \end{array}$$

Here, the element *c*_[*n*-1] _is assigned a maximum value of $X_{\left[n\right]}$(*c *= *c*_[*n*-1]_) = 1. This value represents an absolute certainty that *c*_[*n*-1] _should be excluded from the selection of the device behavior *c*_[*n*] _as stated in Equation (2). Conversely, all other elements share the minimum value $X_{\left[n\right]}$(*c *≠ *c*_[*n*-1]_) = 0, which represents absolute uncertainty about their possibility of exclusion from the selection of *c*_[*n*]_. Thus, $X_{\left[n\right]}$(*c*), which may only take values in the range [0, 1], constitutes a numerical representation of the certainty of exclusion of a given outcome *c *∈ *C *from the selection of *c*_[*n*]_. In other words, $X_{\left[n\right]}$(*c*) may be used to describe a range of assumptions (from weak $X_{\left[n\right]}$(*c*) ≃ 0 to strong $X_{\left[n\right]}$(*c*) ≃ 1) regarding the unsuitability of outcomes in the choice *c*_[*n*]_. This function will be termed the *spatial exclusion mask *of *c*_[*n*]_.

The representation of the NAK principle in Equation (2) by means of the spatial exclusion mask $X_{\left[n\right]}$(*c*) may initially seem unnecessary. However, as it will be demonstrated in the following sections, this mask introduces a framework for the numerical representation of contextual knowledge that may be used to optimize the choice *c*_[*n*] _in response to a single binary event ϵ.

#### Spatial assumptions

In any typical access problem, it is expected that the set of outcomes required to control a device may be numerically arranged in a domain where the distance between similar outcomes is shorter than the distance between dissimilar ones. In that case, outcomes in the neighborhood of *c*_[*n*-1] _would be expected to resemble *c*_[*n*-1]_. This expectation has an important implication in the definition of the spatial exclusion mask $X_{\left[n\right]}$(*c*) because it suggests that all those outcomes near (i.e., similar to) *c*_[*n*-1] _should also be given high (i.e., $X_{\left[n\right]}$(*c*) ≃ 1) values of exclusion from the selection of the outcome *c*_[*n*]_. This is because it may be assumed that outcomes in the neighborhood of *c*_[*n*-1] _are too similar to *c*_[*n*-1] _to cause a significant change in the behavior of the device. This assumption, however, is not as certain as the fundamental principle in Equation (2), because it is not directly implied by the event ϵ. Moreover, the certainty of this assumption should be lower for outcomes that are far apart from *c*_[*n*-1] _than for outcomes that are closer to *c*_[*n*-1]_. Thus, a suitable spatial exclusion mask $X_{\left[n\right]}$(*c*) representing these assumptions may be:

(4) $$X_{\left[n\right]}(c)=\{\begin{array}{ll} 1-\frac{r}{\alpha_{s}} & \text{if }r\leq \alpha_{s}\\ 0 & \text{if }r>\alpha_{s} \end{array}$$

where *r *=|*c *- *c*_[*n*-1] _| is the distance between a given outcome *c *∈ *C *and the outcome *c*_[*n*-1] _∈ *C *preceding the *n*-th event ϵ. In turn, *α*_*s *_is a positive integer used to define the support boundaries *c*_[*n*-1] _± *α*_*s *_of $X_{\left[n\right]}$(*c*). The bottom trace in Figure 3 depicts the updated spatial exclusion mask defined in Equation (4). Note that in the limit *α*_*s *_→ 0, Equation (4) will become Equation (3) as depicted by the top trace in Figure 3.

Evidently, the introduction of the exclusion mask $X_{\left[n\right]}$(*c*) suggests that the best choice of *c*_[*n*] _will be the element *c *∈ *C *that minimizes $X_{\left[n\right]}$(*c*) (i.e., *c*_[*n*] _= argmin $X_{\left[n\right]}$(*c*)). In both cases presented (Figure 3), there is more than one element *c *that fulfills this condition, thus, the selection of *c*_[*n*] _is still ambiguous. However, note that the updated mask $X_{\left[n\right]}$(*c*) described in Equation (4) reduces the number of eligible outcomes *c *∈ *C *to those that lie beyond the support limits *c*_[*n*-1] _± *α*_*s *_of the spatial exclusion mask. In fact, if *α*_*s *_is large enough, a unique solution may be found. The significance of this reduction in the number of eligible outcomes for the choice *c*_[*n*] _will be evident in later discussions. In the meanwhile, note that any function $X_{\left[n\right]}$(*c*) with support limits *c*_[*n*-1] _± *α*_*s *_that decreases monotonically from *c*_[*n*-1] _to *c*_[*n*-1] _± *α*_*s*_, may be used to represent the spatial assumptions described above.

#### Temporal assumptions

The spatial exclusion mask $X_{\left[n\right]}$(*c*) in Equation (4) represents a series of assumptions, with varying degrees of certainty, that outcomes in the spatial neighborhood of *c*_[*n*-1] _should not be eligible in the selection of the subsequent device behavior *c*_ [*n*]_. Similarly, these assumptions may be extended, starting with the outcome *c*_[*n*-1]_, back in time throughout the past history {*c*_[*n*-2]_, *c*_[*n*-3]_, *c*_[*n*-4]_,...} of selected outcomes. Thus, as in the spatial case, outcomes in the temporal neighborhood of *c*_[*n*-1] _(i.e., immediately preceding *c*_[*n*-1]_) should also share a high value of exclusion from the choice *c*_[*n*]_, while outcomes that belong to the remote past history of *c*_[*n*-1] _should be assigned lower values. This is because we may assume that if the recently chosen outcome *c*_[*n*-1] _has already been excluded, there is a high level of certainty that this outcome will not be desired in the near future. However, over time, this outcome should be made available. Evidently, extending this assumption through time requires a memory process that enables the storage of historical information on all outcomes preceding the *n*-th event ϵ. This information must then be available at the time *t*_[*n*]_, when this event occurs, in order to inform the selection of *c*_[*n*]_. The spatial exclusion mask $X_{\left[n\right]}$(*c*), introduced above, cannot be employed for this purpose since it only describes assumptions valid at *t*_[*n*] _without providing any means to describe assumptions associated with the set of past events {*n*-1, *n*-2, *n*-3,...}. Thus, an additional mechanism that enables the incorporation of historical information in the choice *c*_[*n*] _becomes necessary.

### The exclusion estimate

**Figure 4:**
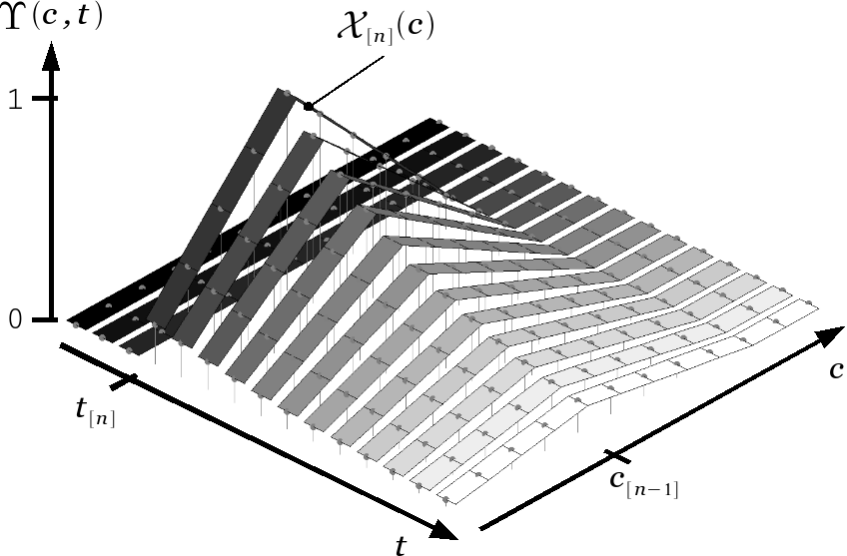
**A discrete time sequence of the viscoelastic deformation of the 1-dimensional domain *C *depicted by function ϒ(*c*, *t*)**. The deformation $X_{\left[n\right]}$(*c*), occurring at time *t*_[*n*]_, experiences a steady decay over time.

Consider the function ϒ(*c*, *t*) depicted in the discrete time sequence presented in Figure 4. This function describes the viscoelastic deformation of the 1-dimensional domain composed of all elements *c *∈ *C*. The figure shows parallel bands representing the state of the domain at regular time intervals. In order to elucidate the progression of time, the bands have been colored from dark to clear corresponding to the transition from older to more recent states of the domain. We will assume ϒ(*c*, *t*) has been left undisturbed for a long time *t *<*t*_[*n*]_allowing it to maintain its natural at state (i.e., ϒ(*c*, *t *<*t*_[*n*]_) = 0 for all possible outcomes *c *∈ *C*). Then, at a given time *t *= *t*_[*n*]_, the domain is subject to a deformation $X_{\left[n\right]}$(*c*) as depicted by the bottom trace in Figure 3. By definition, the viscoelastic deformation process would allow ϒ(*c*, *t*) to recover its natural state. However, as depicted by subsequent bands, this will only happen gradually over time.

The sequence in Figure 4 depicts the temporal memory effect inherent to the mechanical property of viscoelasticity. Note that this property fulfills the requirements stated in the previous section for the incorporation of temporal assumptions in the choice *c*_[*n*]_. In particular, in the context of asynchronous access, the deformation ϒ(*c*, *t*) subject to consecutive disturbances $X_{\left[n\right]}$(*c*), would allow recent assumptions $X_{\left[n\right]}$(*c*) (i.e., those in the temporal neighborhood of *c*_[*n*-1]_) to be assigned higher values of exclusion than former ones. In other words, the function ϒ(*c*, *t*) may be used to record the the full set of historical assumptions represented by all spatial exclusion masks $\left\{X_{\left[n-1\right]}(c),X_{\left[n-2\right]}(c),X_{\left[n-3\right]}(c)...\right\}$ preceding the *n*-th event ϵ. A simple recursive algorithm may be used to represent this process.

Let Δ*t *be the period between the time *t*_[*n*] _of the *n*-th event ϵ and the time *t*_[*n*-1] _of the preceding event, that is

(5) Δ*t *= *t*_[*n*] _- *t*_[*n*-1]_

and ϒ_[*n*]_(*c*) the function ϒ(*c*, *t*) evaluated at time *t*_[*n*]_, that is

(6) ϒ_[*n*]_(*c*) = ϒ(*c*, *t *= *t*_[*n*]_)

The spatial and temporal assumptions previously introduced may then be represented, simultaneously, as the occurrence of disturbances $X_{\left[n\right]}$(*c*) on ϒ_[*n*]_(*c*) with viscoelastic decay $H_{\left[n\right]}$(Δ*t*)

(7) $${ϒ}_{\left[n\right]}(c)=H_{\left[n\right]}(\Delta t){ϒ}_{\left[n-1\right]}(c)+X_{\left[n\right]}(c)\{1-H_{\left[n\right]}(\Delta t){ϒ}_{\left[n-1\right]}(c)\}$$

We will refer to ϒ_[*n*]_(*c*) in Equation (7) as the *exclusion estimate *of the current choice *c*_[*n*]_. Note that, ϒ_[*n*]_(*c*) is defined recursively in terms of the exclusion estimate ϒ_[*n*-1]_(*c*) of the previous choice *c*_[*n*-1]_. All functions ϒ, X and H are constrained to the range [0, 1]. The function $H_{\left[n\right]}$(Δ*t*), used to apply a viscoelastic decay on ϒ_[*n*-1]_(*c*), should decrease monotonically with increasing values of Δ*t*. A suitable choice for $H_{\left[n\right]}$(Δ(*t*) may thus be the family of functions

(8) $$H_{\left[n\right]}(\Delta t)=e^{-\Delta t/\tau }$$

where *τ *is a time constant always greater than zero. The exponential decay described in Equation (8) derives from the behavior of real viscoelastic systems such as the discharge of an electric capacitor or the restoration of a mechanical shock absorber [17]. In all these cases, the constant *τ *is termed the *viscoelastic constant *and it is directly proportional to the duration of the viscoelastic restoration of ϒ_[*n*]_(*c*).

Note that $X_{\left[n\right]}$(*c*) and $H_{\left[n\right]}$(Δ*t*) are weighting functions acting on the spatial and temporal domains, respectively, of the exclusion estimate ϒ_[*n*]_(*c*). While the spatial exclusion mask $X_{\left[n\right]}$(*c*) ensures that outcomes similar to *c*_[*n*-1] _are excluded from the choice *c*_[*n*]_, the function $H_{\left[n\right]}$(Δ*t*) ensures that recent exclusion estimates ϒ(*c*, *t*) are remembered while old ones are forgotten. Thus, $H_{\left[n\right]}$(Δ*t*) is our *temporal exclusion mask*. Note that the support of $H_{\left[n\right]}$(Δ*t*) is defined for values in the range [0, *α*_*t*_] with *α*_*t *_> 0. In the case of the family of functions in Equation (8), *α*_*t *_= ∞.

The definition of the exclusion estimate ϒ_[*n*]_(*c*) in Equation (7), which now integrates spatial and temporal assumptions, suggests that the best possible choice of *c*_[*n*] _should be the element *c *∈ *C *that minimizes ϒ_[*n*]_(*c*). Thus,

(9) *C*_[*n*] _= argmin ϒ_[*n*]_(*c*)

**Figure 5:**
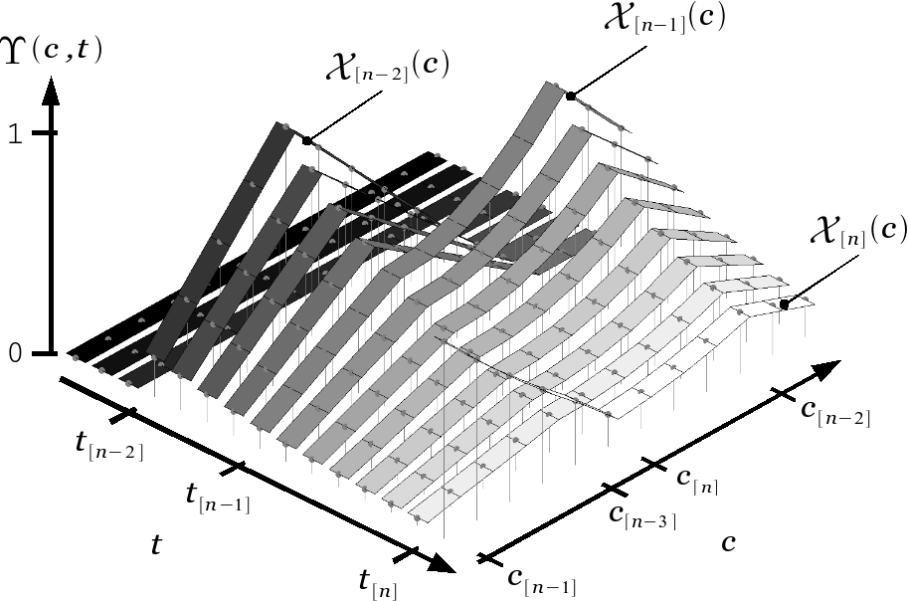
**Discrete time sequence of the evolution of the exclusion estimate ϒ(*c*, *t*) according to Equation (9)**. Three consecutive events ϵ are presented at different intervals.

Figure 5 shows a discrete time sequence of the evolution of ϒ(*c*, *t*), according to Equation (9), where three different events ϵ occur at consecutive times. Note that it has taken only two events ϵ, with corresponding exclusion masks $X_{\left[n-2\right]}$(*c*) and $X_{\left[n-1\right]}$(*c*), for ϒ(*c*, *t*) to converge from a state of absolute uncertainty for *t *<*t*_[*n*-2]_, to a unique solution *c*_[*n*-1] _at *t *= *t*_[*n*-1]_.

Equation (9) summarizes the decision process proposed for the asynchronous selection of a new device outcome *c*_[*n*] _∈ *C *in response to a single binary event ϵ, consisting, in our example of single-switch access, of an intentional button press. Thanks to the assumptions incorporated in $X_{\left[n\right]}$(*c*) and $H_{\left[n\right]}$(Δ*t*), the number of eligible device outcomes in the choice *c*_[*n*] _is significantly reduced. In fact, with the appropriate parameters, Equation (9) will consistently converge to a unique solution soon after the interaction between the user and the device under control is initiated.

The process for asynchronous access presented here incorporates a number of desirable properties that make it easy to implement and adaptable to a wide variety of contexts. Among these properties are:

• There are no restrictions on the time at which a particular event ϵ may occur. For users, this translates into the ability to respond immediately to a change in their intentions or an unexpected external disturbance on the device under control.

• The recursive nature of the exclusion estimate ϒ_[*n*]_(*c*) eliminates the need for the implicit calculation of the effects of the set of historical assumptions $\left\{X_{\left[n-1\right]}(c),X_{\left[n-2\right]}(c),X_{\left[n-3\right]}(c),...\right\}$ on the selection of *c*_[*n*]_, thus reducing the processing power and memory storage capacity required for the implementation of the proposed method for asynchronous access.

• There is no limit on the number *κ *of outcomes *C *that may be made available to the user through this method. In fact, the set *C *may be defined as a continuous interval of all possible real valued outcomes *c *∈ [*c*_*min*_, *c*_*max*_], where *c*_*min *_and *c*_*max *_are the lower and upper boundaries of *C*, respectively. Evidently, in this case, *κ *= ∞.

### Summary

1. According to Equation (2), when the *n*-th event ϵ occurs, the device outcome *c*_ [*n*] _must be different from the outcome *c*_ [*n*-1] _immediately preceding it. In other words, there is absolute certainty that *c*_ [*n*-1] _should be excluded from the selection of *c*_ [*n*]_. Thus, the event ϵ, which represents a voluntary, user-prompted change in the interface, should be employed by users as an error indicator. This requires users to generate events ϵ every time the behavior of the device is inconsistent with their intentions.

2. Even though the exclusion principle in Equation (2) is the only knowledge implied, with absolute certainty, by the occurrence of event ϵ, it is also possible to assume, although with a lower degree of certainty, that behaviors similar to *c*_[*n*-1] _should also be excluded from the selection of *c*_[*n*]_. This assumption is defined by the spatial exclusion mask $X_{\left[n\right]}$(*c*), a function with values in the range [0, 1] and support *c*_[*n*-1] _± *α*_*s*_, decreasing monotonically from $X_{\left[n\right]}$(*c *= *c*_[*n*-1]) _= 1 (i.e., the strongest assumption of exclusion) to $X_{\left[n\right]}$(*c *= *c*_[*n*-1] _± *α*_*s*_) (i.e., the weakest assumption of exclusion) as candidate outcomes *c *∈ *C *become decreasingly similar to *c*_[*n*-1]_.

3. It may also be assumed that device outcomes resulting from recent selections (i.e., immediately preceding the *n*-th event ϵ), should be excluded from the selection of *c*_[*n*]_, while outcomes that belong to the remote past of *n *should become eligible. The incorporation of this assumption is made possible through the introduction of the exclusion estimate ϒ_[*n*]_(*c*) and the temporal exclusion mask $H_{\left[n\right]}$(Δ*t*), where Δ*t *is, according to Equation (5), the period between the time *t*_[*n*] _of the *n*-th event and the time *t*_[*n*-1] _of its predecessor. According to Equation (7), the exclusion estimate ϒ_[*n*]_(*c*), which is recursively defined in terms of the exclusion estimate ϒ_[*n*-1]_(*c*) of the preceding event, acts as a viscoelastic domain storing the set of historical deformations $\left\{X_{\left[n\right]}(c),X_{\left[n-1\right]}(c),X_{\left[n-2\right]}(c),...\right\}$ subject to a viscoelastic decay described by the temporal exclusion mask $H_{\left[n\right]}$(Δ*t*). Thus, $H_{\left[n\right]}$(Δ*t*) must decrease monotonically from $X_{\left[n\right]}$(Δ*t *= 0) = 1 to $H_{\left[n\right]}$(Δ*t *= ∞) = 0. Note that the functions $X_{\left[n\right]}$(*c*) and $H_{\left[n\right]}$(Δ*t*) act as weighting masks on ϒ_[*n*]_(*c*) updating the certainty of exclusion, from the choice *c*_[*n*]_, for every candidate outcome *c *∈ *C*, according to reasonable spatial and temporal assumptions, respectively.

4. Once the exclusion estimate ϒ_[*n*]_(*c*) is calculated, it will be possible to make an informed decision regarding the best possible choice of *c*_[*n*] _∈ *C *according to Equation (9).

## Implementing the proposed method

In order to successfully implement the method for asynchronous binary access presented above, some additional considerations are required.

### Initialization

Note that the decision process described in Equation (9) does not specify the characteristics of the exclusion estimate ϒ_[0]_(*c*) before the first (i.e., *n *= 1) event ϵ is generated by the user. In fact, there is no information regarding the value of the initial device outcome *c *_[0] _either, and without this knowledge, the recursive process described in Equation (7) may not be initialized. Thus, even before the user initiates interaction with the device, a virtual selection *c*_[0] _must be made. Similarly to the case where the concept of viscoelasticity was first introduced, we may assume that before the first user-prompted event (i.e., *t *<*t*_[1]_) the exclusion estimate had been left undisturbed for a long time, thus allowing it to recover its natural flat state. That is, ϒ_[0]_(*c*) = 0 for all outcomes *c*_[0] _= *c *∈ *C*. Moreover, since all values *c *∈ *C *fulfill the condition in Equation (9), we would then be obliged to draw *c*_[0] _from a uniform distribution of *C*. Consequently, this random selection of *c*_[0] _may be used to initialize the decision process Equation (9). Note that it is not necessary to communicate the virtual choice *c*_[0] _to the device under control. Thus, the device may remain undisturbed until after the first user-prompted event ϵ occurs. In this case, the virtual choice *c*_[0] _will only be used to obtain the first exclusion mask $X_{\left[1\right]}$(*c*) at *t*_[1]_, enabling the calculation of the estimate ϒ_[1]_(*c*). The resulting outcome *c*_[1] _will then be the first to affect the device's behavior. From the perspective of the user, it will appear that the outcome *c*_[1] _has been drawn randomly from a uniform distribution. However, as explained here, this is only the case for the virtual choice *c*_ [0]_, since, according to Equation (9) *c*_[1] _will be drawn from a more restricted distribution where a subset of the elements *c *∈ *C *(i.e., ~ *c*_[0] _± *α*_*s*_) have already been excluded.

An alternative (and, in fact, more useful) procedure consists of initializing ϒ_[0]_(*c*) with random white noise in the interval of real numbers [0, 1]. This minimizes the probability of having multiple candidates for the virtual choice *c*_[0]_, since it is expected that, after this initialization, ϒ_[0]_(*c*) will present a unique minimum value, which may then constitute the virtual choice *c*_[0]_. The advantage of this method over the one initially proposed, resides in the fact that with the latter, ϒ_[*n*]_(*c*) will more likely converge to a unique solution from the beginning (i.e., *n *= 1) of the interaction. In fact, this also allows for the prediction of future selections of *c*_[*n*] _∈ *C *with a significant degree of confidence.

### Anchorage

If the viscoelastic constant *τ *is too long, or a significant number of events ϵ occur in a short amount of time, the exclusion estimate ϒ_[*n*]_(*c*) may accumulate constant offsets from previous, but still remembered deformations X(*c*). Due to the discretization process inherent to any numerical implementation of the proposed method (e.g., on a computer), this offset accumulation may in fact cause saturation of the exclusion mask ϒ_[*n*]_(*c*). That is, ϒ_[*n*]_(*c*) ≃ 1 for all outcomes *c *∈ *C*. If saturation occurs, the information storage capacity of the exclusion estimate will be completely eliminated, thus, preventing the selection of reasonable outcomes *c*_ [*n*] _derived from the spatial and temporal assumptions introduced before.

In order to prevent the occurrence of saturation, constant offsets must be eliminated at all times from the exclusion estimate ϒ_[*n*]_(*c*). This may be achieved by subtracting the value of ϒ_[*n*]_(*c *= *c*_[*n*]_) from the function ϒ_[*n*]_(*c*). That is

(10) ϒ_[*n*]_(*c*) ⇐ ϒ_[*n*]_(*c*) - ϒ_[*n*]_(*c *= *c*_[*n*]_)

where ϒ_[*n*]_(*c *= *c*_[*n*]_) is the value of ϒ_[*n*]_(*c*) evaluated at the recently obtained outcome *c*_[*n*]_. Evidently, if ϒ_[*n*]_(*c *= *c*_[*n*]_) is already zero, Equation (10) will have no effect on ϒ_[*n*]_(*c*). This process of elimination of the offset of the exclusion estimate ϒ_[*n*]_(*c*) is termed *anchorage*. The process of anchorage has no effect on the decision *c*_[*n*]_, since this decision only depends on the relative exclusion value of a given outcome *c *∈ *C *as compared to the rest of the elements of *C*.

### Algorithm

The following list summarizes the sequential steps required for the implementation of the proposed asynchronous access method.

1. Originally, nothing is known about the intention of the user regarding the behavior of the device. Thus, the exclusion estimate ϒ_[0]_(*c*) may be initialized with white noise in the range [0, 1]. This results in the definition of the virtual choice *c*_ [0] _and the exclusion mask $X_{\left[1\right]}$(*c*), which precede any user interaction and, therefore, any change in the behavior of the device.

2. When the *n*-th intentional binary event ϵ occurs, the period Δ*t *is calculated according to Equation (5) and used to obtain the decay $H_{\left[n\right]}$(Δ*t*) through a suitable function such as Equation (8). Subsequently, the intention estimate ϒ_[*n*]_(*c*) is updated according to Equation (7).

3. The corresponding *n*-th device outcome *c*_ [*n*] _may now be obtained according to Equation (9). This outcome is immediately transmitted to the device which experiences a change in behavior.

4. The exclusion estimate ϒ_[*n*]_(*c*) is anchored according to Equation (10).

5. The exclusion mask $X_{\left[n\right]}$(*c*) is updated to $X_{\left[n+1\right]}$(*c*) through a suitable function such as Equation (4).

6. For subsequent events ϵ, the process is repeated from (ii) above.

In addition, the fundamental spatial and temporal assumptions require their corresponding exclusion masks $X_{\left[n\right]}$(*c*) and $H_{\left[n\right]}$(Δ*t*) to have the following properties:

• The spatial exclusion mask $X_{\left[n\right]}$(*c*) must decrease monotonically from $X_{\left[n\right]}$(*c *= *c*_[*n*-1]_) = 1 to $X_{\left[n\right]}$(*c *= *c*_[*n*-1] _± *α*_*s*_) = 0. The support of this function will be defined in the range [*c*_[*n*-1] _- *α*_*s*_, *c*_ [*n*-1] _+ *α*_*s*_].

• The temporal exclusion mask $H_{\left[n\right]}$(Δ*t*) must decrease monotonically from $H_{\left[n\right]}$(Δ*t *= 0) = 1 to $H_{\left[n\right]}$(Δ*t *= *α*_*t*_) = 0. The support of this function will be defined in the range [0, *α*_*t*_] where *α*_*t *_> 0.

Note that although these assumptions are reasonable given the access problem proposed, there is no limit to the number and/or kind of assumptions that may be incorporated into $X_{\left[n\right]}$(*c*) and $H_{\left[n\right]}$(Δ*t*). For example, one could deliberately exclude a particular outcome *c*_*i *_∈ *C *(i.e., $H_{\left[n\right]}$(*c*_*i*_) = 1) or all events occurring before a certain memory threshold Δ*t*_0 _(i.e., $H_{\left[n\right]}$(Δ*t *< Δ*t*_0_) = 0) in response to some contextual knowledge.

## Evaluation

We have presented a method for asynchronous binary access based on the selection of a particular outcome *c*_[*n*] _that may be immediately transmitted to the device under control in response to the single *n*-th binary event ϵ. However, we have not yet given any consideration to the case when the selected outcome *c*_[*n*] _∈ *C *is inconsistent with the user's intention. This is, in fact, a very likely possibility if we consider that, according to the NAK signaling process previously described, by generating the event ϵ the user is simply requesting a change in the behavior of the device. However, there are no means to specify which of the outcomes *c *∈ *C *will be the most appropriate. Thus, if the outcome *c*_[*n*] _∈ *C *chosen after the *n*-th event ϵ is unacceptable, the user will be required to generate another event ϵ hoping to obtain the desired outcome with the subsequent choice *c*_[*n*+1] _∈ *C*. Users will be required to repeat this process until the behavior of the device is consistent with their intention.

For the typical binary interface user, generating the event ϵ will require some kind of effort. Thus, measuring the number of events ϵ required to reach a particular target outcome *c*_γ _∈ *C *would provide a benchmark for the evaluation of the cost associated with the proposed method. Note, however, that this measure arises from a naturally uncertain (i.e., stochastic) process and thus, may only be described in terms of probability.

Let *N *be the number of intentional binary events ϵ required to reach a series of typical target outcomes *c*_γ _∈ *C*, it is possible to measure the fraction *P *(*N *≤ *X*) of targets *c*_γ _that will require *X *or less events ϵ to be reached. This is known in probability theory as the cumulative distribution function (CDF) of the random variable *N *[18].

**Figure 6:**
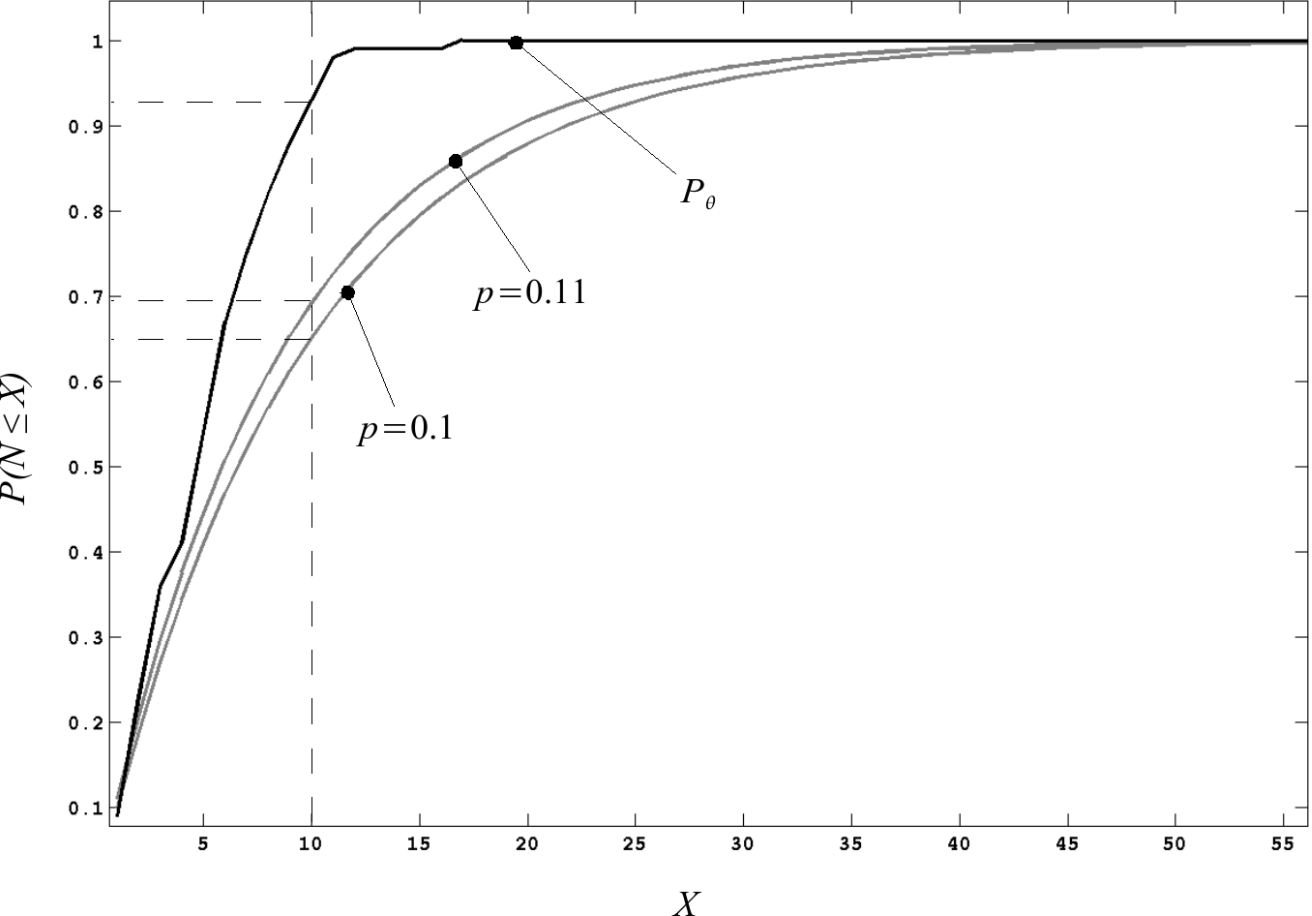
**Cumulative distribution functions obtained from the process of selection of a given outcome *c *∈ *C *subject to a fractional tolerance *σ *= 10% of the full range of *C***. The lower gray trace corresponds to a selection with substitution with probability of success *p *= 0.1. The middle gray trace corresponds to a selection without substitution *p *= 0.11 and the upper black trace corresponds to the proposed method for asynchronous access with parameters *ω *= 0.05, *τ *= 5}.

Figure 6 depicts a sample CDF corresponding to two different processes of selection of a specific outcome *c*, drawn from a uniformly distributed set *C *of size *κ *= 8, in response to consecutive binary events ϵ. Both processes follow a geometric distribution with CDF defined as

(11) *P*(*N *≤ *X*) = 1 - (1 - *p*)^*X*^

where *p *is the probability of making a correct choice for any given attempt.

The lower trace in Figure 6 (i.e., *p *= 0.125) results from a selection with substitution where all outcomes *c *∈ *C *are eligible on every trial *n*. Conversely, the trace with *p *= 0.143 results from a selection without substitution where the outcome *c*_[*n*-1] _is eliminated from the *n*-th trial. This reduction in the number of eligible outcomes in the choice *c*_[*n*] _increases the probability *p *of making a correct choice at any given trial *n*. Thus, as indicated by the dashed lines, with the latter process of selection without substitution, there is a marginal gain in the fraction *P*(*N *≤ *X*) of targets *c*_γ _that may be reached with *X *= 10 or less trials. The process of selection without substitution described above, is identical to the fundamental principle of asynchronous access presented in Equation (2), which describes the selection of a new device outcome *c*_[*n*] _in response to the user-prompted event ϵ. Thus, as demonstrated in Figure 6, the incorporation of the knowledge implied by this principle, translates directly into a reduction of the cost associated with the use of the device (i.e., a reduction in the expected number, *N*, of events ϵ required to reach a target outcome *c*_γ_). Similarly, it would be desirable to evaluate the impact that the additional assumptions $X_{\left[n\right]}$(*c*) and $H_{\left[n\right]}$(Δ*t*), incorporated in the exclusion estimate ϒ_[*n*]_(*c*), may have on the cost of use of the device.

However, the CDF associated with these assumptions is not easy to obtain since it depends on a variety of parameters specific to the particular context of application. Thus, in order to evaluate the performance of the proposed access method, we completed a series of simulations for a select case of device control by a binary interface user.

## Methods

In order to determine the expected impact on the cost of use of a device associated with the proposed method for asynchronous binary access, a Monte Carlo simulation [19] of a simple access task was performed. In this simulated environment, a computer model of a typical user was implemented. This model user was then required to employ a single binary interface in order to select a series of predefined targets *c*_γ _from a set *C *of *κ *= 100 outcomes required to control a device (e.g. the volume of a TV). It was assumed that the main mode of monitoring the behavior of the device by the model user was visual. Thus, as soon as the model user 'observed' that the behavior of the device was inconsistent with the required target, an intentional event ϵ would be generated. A total of 1000 elements *c*_γ _were initially drawn randomly with replacement from *C *in order to establish the predefined sequence of target outcomes that the user was required to select in order to successfully control the device. It is important to note that real access applications are likely to involve sequences of correlated actions rather than independent ones. Thus, our choice of random, uncorrelated targets represents an extreme case likely to constitute a lower boundary of performance for the proposed access method.

Each target in the sequence was presented to the model user until it was reached. Then, the next target in the sequence was presented, and so on. The objective of this simulation was to measure the number *N *of intentional events ϵ that would be required from the user in order to reach each target *c*_γ_. This process was repeated 6 times for a total of 6000 targets per trial. This number was sufficient to quantify the statistical nature of *N *and obtain an estimate of its CDF for each case evaluated.

### Modelling the user

As mentioned before, it was assumed that the model user was able to monitor the behavior of the device under control through visual means. This process would involve a series of delays as a result of the time required by the user to process the visual information and, if necessary, generate the event ϵ. Thus, in order to obtain an accurate model of the visual reaction time *t*_*r*_, which includes both the visual perception and motor reaction times, an initial experiment was performed with a real user. During this experiment, the real user was requested to respond to simple visual stimuli presented on a computer screen. The stimuli consisted of the appearance of a white circle on a black background after random delays of 1 to 3 seconds. The user was instructed to press a button (defined as the event ϵ) immediately after the stimulus (i.e., the white circle) appeared on the screen. The experiment was performed using the open source software package PXLab, which can be used to accurately measure the user's reaction time *t*_*r *_defined as the period from the presentation of the stimulus, to the generation of the intentional event ϵ. A histogram of the reaction times, *t*_*r*_, was obtained with a total of 100 trials. This histogram was used to represent the model user in the Monte Carlo simulations introduced above. Thus, for each event ϵ, a reaction time, *t*_*r*_, was randomly drawn from the histogram. The expected value $\bar{t_{r}}$ of this user's reaction time was ~213 ms, which is consistent with previous research on the topic [20]. Thus, it may be assumed that the statistical model, represented by the histogram obtained, was an accurate estimate of user behavior incorporating the stochastic nature of the interaction between a real user and a device.

### Cases for evaluation

According to the proposed method of asynchronous control, there are few restrictions to the definitions of the exclusion masks $X_{\left[n\right]}$(*c*) and $H_{\left[n\right]}$(Δ*t*). As a result, there is an infinite number of functions that comply with the basic requirements of both of these functions. We will focus on the evaluation of a single family of functions for each of the exclusion masks defined. These functions have already been introduced and correspond to some of the simpler sets of assumptions that may be made in compliance with the necessary requirements for the linear spatial exclusion mask $X_{\left[n\right]}$(*c*) in Equation (4) and the exponential temporal exclusion mask $H_{\left[n\right]}$(Δ*t*) in Equation (8).

Each case for evaluation was defined by a set, $\underset{˜}{\theta }$, of three parameters:

• The tolerance *σ *of the choice *c*_[*n*]_. This parameter was used to define a boundary around the target outcome *c*_*γ *_within which the choice *c*_[*n*] _was considered acceptable. In other words, the target outcome *c*_*γ *_was reached if

(12) $$\left|\frac{c_{\left[n\right]}-c_{\gamma }}{c_{max}-c_{min}}\right|<\frac{\sigma }{2}$$

• The width *ω *= *α*_*s *_·(*c*_*max *_- *c*_*min*_)^-1 ^of the linear spatial exclusion mask $X_{\left[n\right]}$(*c*). This parameter specified the fraction of the full length of *C *defining the support boundaries of $X_{\left[n\right]}$(*c*) as defined in Equation (4).

• The viscoelastic constant *τ *of the temporal exclusion mask $H_{\left[n\right]}$(Δ*t*) as defined in Equation (8). This parameter defined the expected size, in seconds, of the memory window of the exclusion estimate ϒ_[*n*]_(*c*).

Table 1 shows the admissible and selected test values for all parameters in $\underset{˜}{\theta }$. In the context of this simulation, *σ *represented a requirement of the particular device under control while *ω *and *τ *characterized the algorithms employed to enable access to the device. In other words, the simulation evaluated the performance of a number of algorithms described by *ω *and *τ *on the solution of particular access problems with a *σ *requirement. The test values for the parameter set $\underset{˜}{\theta }$ were selected according to generalized but expected contexts of human-machine interaction, within relatively broad intervals. Thereore, this simulation may only be used to characterize the general properties of the proposed method for asynchronous access. In order to evaluate the performance of this method in specific applications, more complete simulations incorporating the appropriate parameters are necessary.

**Table 1:** Admissible and test values for the parameter set $\underset{˜}{\theta }$ evaluated

Parameter	Admissible Values	Test Values
*σ*	[0, 1]	{0.05, 0.1, 0.15, 0.20}
*ω*	[0, ∞]	[0.02, 0.2] in regular increments of 0.01
*τ*	[0, ∞]	*τ *= *e*^*z*^ where *z *∈ [-2.5, 3.75] in regular increments of 0.25

In total, 1976 sets $\underset{˜}{\theta }$ = {*σ*, *ω*, *τ*} were evaluated, and, as mentioned before, each set consisted of 6000 separate trials where the model user was requested to reach a specific target *c*_*γ *_using a single binary interface and the proposed method for asynchronous access.

### Performance measure

According to Figure 6, for every possible value of *X*, the value of the fraction *P*(*N *≤ *X*) will be higher for algorithms that reduce the cost of access when compared to a simple random selection with substitution. Furthermore, the higher the value *P*(*N *≤ *X*), the more cost-effective the associated algorithm will be. Therefore, if the differences between the corresponding values *P*(*N *≤ *X*) for a given algorithm and a simple random selection are accumulated over *X*, it will be possible to obtain an overall score for each of the cases evaluated. We defined the overall score Γ($\underset{˜}{\theta }$) as

(13) $$\Gamma (\theta )=\sum_{X=1}^{\infty }P_{\underset{˜}{\theta }}(N\leq X)-\sum_{X=1}^{\infty }1-{\left(1-\sigma \right)}^{X}$$

where $P_{\underset{˜}{\theta }}$(*N *≤ *X*) is the CDF obtained for a particular set $\underset{˜}{\theta }$ = {*σ*, *ω*, *τ*}. Note that Γ is a relative measure of performance with reference to a process of random selection with substitution subject to the given tolerance *σ *. This latter process is captured by the second term in Equation (13) and defined in Equation (11). In the limit *X *→ ∞, both terms of the subtraction tend to 1 thus, in practice, it is only necessary to consider a sufficiently large number for *X*. For example, in the case presented in Figure 6, the value *X *= 40 would be an appropriate limit for the summations.

According to Equation (13), positive values of Γ($\underset{˜}{\theta }$) indicate lower usage costs. Thus, in order to optimize the cost, Γ($\underset{˜}{\theta }$) must be maximized. Conversely, negative values of Γ($\underset{˜}{\theta }$) would indicate a disastrous performance (i.e., even worse than a random guess). Finally, a value Γ($\underset{˜}{\theta }$) = 0 would indicate similar performance between a random guess and the proposed algorithm with the particular parameter set $\underset{˜}{\theta }$. However, in such cases, the additional complexity of the proposed method would not justify its application. Thus, these sets should also be avoided.

## Results and discussion

Figure 6 presents the CDF resulting from a single case $\underset{˜}{\theta }$ = {*σ *= 0.1, *ω *= 0.05, *τ *= 5} as compared to the CDF obtained from a simple selection from a uniform distribution, with (*p *= 0.1) and without (*p *= 0.11) substitution, subject to the same tolerance requirement *σ *= 0.1. Note that, for a given value *X *= 10, the proposed method provides an advantage in excess of 20% in the fraction $P_{\underset{˜}{\theta }}$(*N *≤ *X*) of trials completed.

Furthermore, this method reaches a maximum value $P_{\underset{˜}{\theta }}$(*N *≤ *X*) = 1 with less than half the events ϵ required by the other selection processes. In other words, the proposed method demands over 50% less effort from the user. With the parameter values specified above, the value of relative performance as defined in Equation (13) was Γ($\underset{˜}{\theta }$) ≈ 4.25

**Figure 7:**
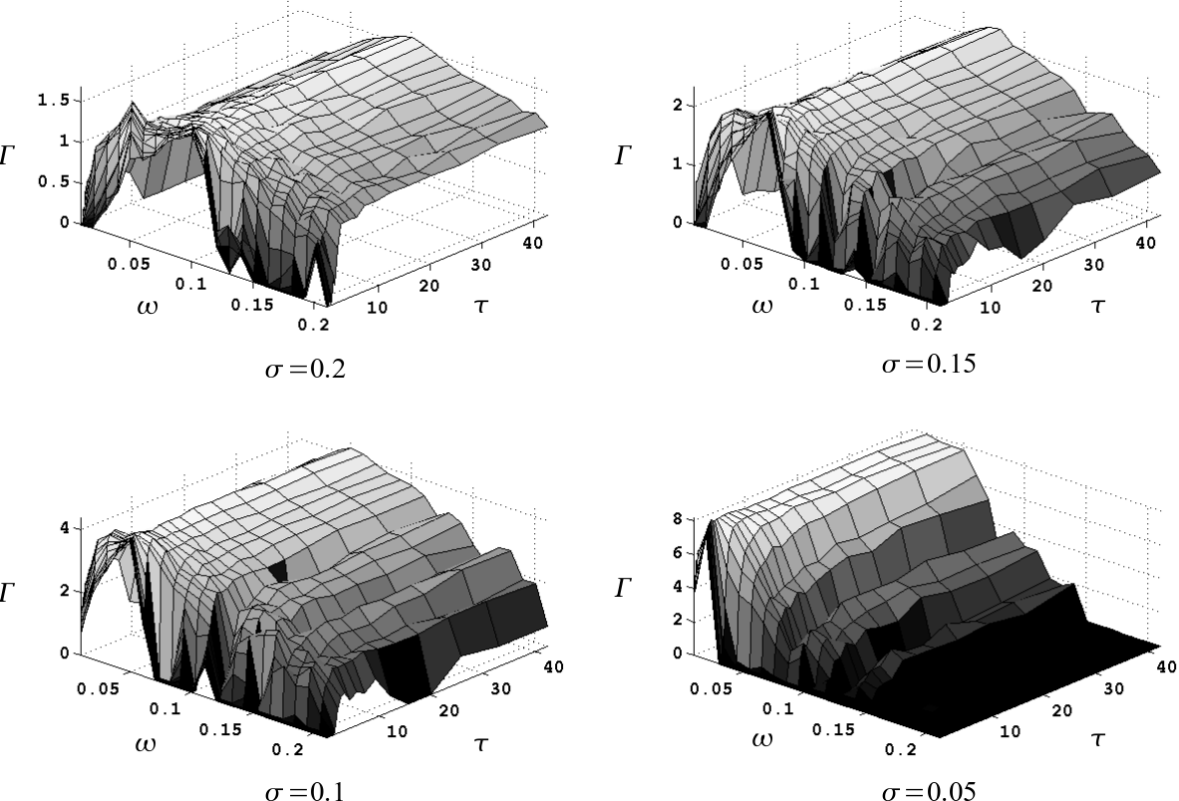
**Relative performance Γ for all sets of parameters $\underset{˜}{\theta }$ = {*σ*, *ω*, *τ*} evaluated**. The viscoelastic constant *τ *has a negligible effect beyond ~10 $\bar{t_{r}}$. Furthermore, with lower values of tolerance *σ*, the set of acceptable parameters {*ω*, *τ*} is reduced. However, the relative gain Γ may be significantly larger than that obtained with greater tolerances.

Figure 7 shows all 1976 cases evaluated. Cases with relative performance values lower than zero were plotted here as Γ($\underset{˜}{\theta }$) = 0. Note that only a small fraction of cases exhibited this unacceptable value of performance.

As mentioned before, the results of this simulated experiment must be interpreted with care. In particular, we have identified three significant concerns i) a real application is likely to involve other cognitive processes in addition to the simple visual reaction time used here to model the user, ii) the control of a real device is likely to involve a series of correlated targets instead of the independent ones proposed in our experiment, and iii) users can fail trying to activate the interface and cause a delay, but, worst, the user can involuntarily activate it even if (s)he is happy with the current choice.

Regarding the first concern, the reaction time of the user will likely be increased in real applications, stretching the relative performance measure Γ in the *τ *axis. However, as shown in Figure 7, for all cases, the influence of *τ *is negligible beyond approximately 10 times the expected user reaction time $\bar{t_{r}}$. Thus, if a sufficiently large *τ *> 10·$\bar{t_{r}}$ is chosen, the performance of the algorithm will not be significantly impacted.

Moreover, with the proposed asynchronous access method, the user must only determine whether the device is behaving erroneously or not. In most cases, this should be obvious to them. Therefore, the actual reaction time may not be significantly longer than the simple visual reaction time considered in this experiment.

In terms of the second concern, the use of uncorrelated targets drawn from a uniform distribution has likely resulted in a lower boundary of performance for the experiments carried on here. In other words, the proposed method for asynchronous access is expected to have a better performance in a real application.

This is because real applications are likely to be composed of correlated targets whose spatial and temporal relationships are approximated by the basic assumptions incorporated in X and H, respectively.

Finally, in cases where the user involuntarily rejects correct behaviors, (s)he will be forced to activate the interface a few more times in order to reach, once more, such behavior. However, it is important to note that, for the proposed example, the algorithm's performance will still be subject to the pattern reported in figure 6 even though the correct behavior will be placed at the end of the queue immediately after an involuntary rejection. That is, it will still take *X *≃ 18 or less interface activations to reach the target again. On average, however, this process will take longer than with a random selection. Thus, for settings in which the probability of false-positives (i.e. involuntary rejections) is high, the performance of the algorithm may be significantly compromised. The reasons for increased false-positive rates in a specific application depend not only on the user's ability to maintain a particular selection, but also on the performance of the binary interface itself. In order to mitigate the incidence of false-positives, a variety of strategies can be used ranging from adaptations of the physical environment (including the interface) to the implementation of digital filters that disambiguate the user's intention. Due to the complexity and interdependence among the different factors that may influence performance in a specific context, this issue must be studied on a case-by-case basis. We will explore in detail the real impact of false-positives and its potential mitigation in further studies involving real users that attempt to control complex appliances using a binary interface in combination with the proposed algorithm.

From the results presented above, one may also observe that the number of parameters {*ω*, *τ*} (i.e., the width of the spatial exclusion mask and the viscoelastic constant, respectively), which result in maximum relative performance Γ, increases with the tolerance *σ *of the particular application. Conversely, the maximum relative gain Γ, obtained with higher tolerances *σ*, is reduced in comparison to cases where the tolerance is small. Thus, for instance, while a wider range of parameters {*ω*, *τ*} is acceptable in the case *σ *= 0.2, the maximum gain obtained with optimal parameters {*ω*, *τ*} in the case *σ *= 0.05 is significantly higher. This phenomenon represents the main trade-off of the proposed method. Thus, in principle, the proposed asynchronous access method may be used to determine the behavior of a device with any degree of precision; however, higher precision will require more rigorous fine tuning of the algorithm parameters {*ω*, *τ*}.

In all cases, maximum values of performance were reached when *ω *= *σ*/2. This actually corresponds to the cases where the exclusion mask $X_{\left[n\right]}$(*c*), characterized by *ω*, corresponded with the actual requirements of the application summarized by the tolerance *σ*. Evidently, if the tolerance *σ *of a particular application is known, the optimal value *ω *= *σ*/2 may be immediately set. However, in a real application, this tolerance may not be easily identified. Furthermore, tolerance is likely to depend on the control priorities of each user in a particular application. Thus, for example, within the maximum tolerance for the execution of a particular task, some users may be more willing to accept errors than others. Nevertheless, the wide variety of arrangements available through the proposed access method, allows for its adaptation to virtually any type of user.

### A sample application

**Figure 8:**
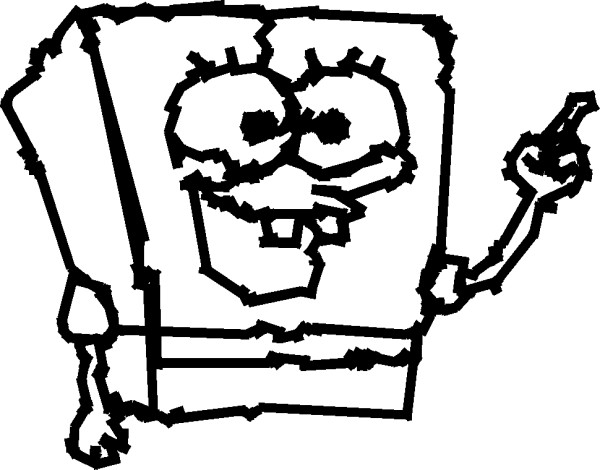
**Sample drawing made by a minimal interface user by means of the one-button doodler on-line software application **[21]. A particular implementation of the proposed method, with spatial exclusion mask $X_{\left[n\right]}$(*c*) defined by Equation (14), allowed this user to access a total of 180 different angles, allowing a pointer to follow the trajectory defined by the depicted trace.

In order to demonstrate the relevance of the proposed asynchronous access method in the control of real appliances, we implemented a single-switch drawing application termed the *one-button doodler *[21], this on-line tool allows minimal interface users to create "free-hand" drawings using only the left button of a computer mouse or a single keyboard key. Figure 8 depicts a sample drawing made by a minimal interface user by means of this software application. A particular implementation of the proposed method, with spatial exclusion mask $X_{\left[n\right]}$(*c*) defined by Equation (14), allowed this user to access a total of 180 different angles, allowing a pointer to follow the trajectory defined by the depicted trace. Note that, given the angular nature of the domain under control, the mask introduced here was more appropriate for this application than the mask defined in Equation (4) above.

(14) $$X_{\left[n\right]}(c)=\frac{1}{2}[1+\cos(r)]$$

We have previously reported that, in order to minimize the delay between the user's action and the device's response, the outcome *c*_[*n*] _is transmitted to the device immediately after it is selected. However, in recent experiments involving real users, it has been evident that this immediacy is not as important as the ability to select the intended behaviour with high accuracy. Thus, in some cases, users prefer to have some time to reject the most recent selection proposed by the algorithm. Furthermore, since the algorithm becomes highly predictable soon after the interaction with the user has been initiated, it is possible to display a list of suggested behaviors that will follow the most recent selection. When available, this additional information can improve accuracy significantly. Full results of these and other studies involving real users will be reported in subsequent publications.

## Conclusion

A novel method of asynchronous binary access has been proposed. This method translates consecutive intentional changes, executed by users of binary interfaces at irregular intervals, into increasingly accurate estimates of their intention. With this method, users are required to employ their interfaces only when the device under control behaves erroneously. When this happens, an algorithm that incorporates simple spatial and temporal assumptions, regarding all possible device outcomes, is used to obtain an informed estimate of the best possible outcome that the device should present next. This algorithm is based on the mechanical deformation of a viscoelastic space that may store the set of historical assumptions preceding any intentional event performed by the user. The theoretical evaluation of this method resulted in two significant conclusions:

1. The proposed method may be used with binary interfaces to asynchronously access devices with any number of potential outcomes and,

2. this method may be optimized through the particular choice of the spatial and temporal exclusion masks X and H, according to the particular requirements and contextual circumstances of each application.

## Authors' contributions

JS designed the asynchronous selection algorithm, the software tools and data structures for the experiments. JS proposed the initial design of experiments, executed them, and analyzed and interpreted the data. JS worked on the initial draft of the manuscript. JT, TC and AM advised upon the design and coordination of the study, experiments and data analysis, and multiple revisions of the manuscript. All authors read and approved the final version of the manuscript.

## Competing interests

The authors declare that they have no competing interests.
